# Heterogeneous Network With Multiview Path Aggregation: Drug–Target Interaction Prediction Study Design

**DOI:** 10.2196/74974

**Published:** 2025-10-02

**Authors:** Haixue Zhao, Kui Yao, Yunjiong Liu, Chao Che, Lin Tang

**Affiliations:** 1 Key Laboratory of Advanced Design and Intelligent Computing, Ministry of Education, Dalian University, Dalian, China; 2 School of Software Engineering, Dalian University, No. 10 Xuefu Street, Dalian Economic and Technological Development Zone, Liaoning, Dalian, 116622, China, 86 18940965015; 3 Institute of Artificial Intelligence, School of Informatics, Xiamen University, Xiamen, China; 4 National Institute of Diagnostics and Vaccine Development in Infectious Diseases, National Innovation Platform for Industry-Education Integration in Vaccine Research, NMPA Key Laboratory for Research and Evaluation of Infectious Disease Diagnostic Technology, Xiamen University, Xiamen, China; 5 State Key Laboratory of Vaccines for Infectious Diseases, Xiang An Biomedicine Laboratory, School of Public Health, Xiamen University, Xiamen, China

**Keywords:** drug-target interactions, drug repurposing, heterogeneous networks, graph neural networks

## Abstract

**Background:**

Drug–target interaction (DTI) prediction is crucial in drug repositioning, as it can significantly reduce research and development costs and shorten the development cycle. Most existing deep learning–based approaches employ graph neural networks for DTI prediction. However, these approaches still face limitations in capturing complex biochemical features, integrating multilevel information, and providing interpretable model insights.

**Objective:**

This study proposes a heterogeneous network model based on multiview path aggregation, aiming to predict interactions between drugs and targets.

**Methods:**

This study employed a molecular attention transformer to extract 3D conformation features from the chemical structures of drugs and utilized Prot-T5, a protein-specific large language model, to deeply explore biophysically and functionally relevant features from protein sequences. By integrating drugs, proteins, diseases, and side effects from multisource heterogeneous data, we constructed a heterogeneous graph model to systematically characterize multidimensional associations between biological entities. On this foundation, a meta-path aggregation mechanism was proposed, which dynamically integrates information from both feature views and biological network relationship views. This mechanism effectively learned potential interaction patterns between biological entities and provided a more comprehensive representation of the complex relationships in the heterogeneous graph. It enhanced the model’s ability to capture sophisticated, context-dependent relationships in biological networks. Furthermore, we integrated multiscale features of drugs and proteins within the heterogeneous network, significantly improving the prediction accuracy of DTIs and enhancing the model’s interpretability and generalization ability.

**Results:**

In the DTI prediction task, the proposed model achieves an AUPR (area under the precision-recall curve) of 0.901 and an AUROC (area under the receiver operating characteristic curve) of 0.966, representing improvements of 1.7% and 0.8%, respectively, over the baseline methods. Furthermore, a case study on the KCNH2 target demonstrates that the proposed model successfully predicts 38 out of 53 candidate drugs as having interactions, which further validates its reliability and practicality in real-world scenarios.

**Conclusions:**

The proposed model shows marked superiority over baseline methods, highlighting the importance of integrating heterogeneous information with biological knowledge in DTI prediction.

## Introduction

### Background

Drugs play a crucial role in treating diseases by interacting with multiple targets and modulating their functions. Accurately predicting drug–target interactions (DTI) is essential for understanding drug mechanisms [1], discovering new targets, and facilitating drug repositioning. Traditional methods rely heavily on large amounts of labeled data and often suffer from label noise in negative samples. In recent years, positive-unlabeled learning has been effectively applied to alleviate this issue [2]. Meanwhile, self-supervised learning strategies integrated with heterogeneous biomedical networks have improved the accuracy of DTI prediction by leveraging multimodal information [3]. With the rapid advancement of large language models (LLM), their applications in the biomedical domain have expanded significantly. Due to their powerful sequence representation and semantic understanding capabilities, LLMs are driving DTI prediction methods toward a higher level of performance [4].

Currently, DTI prediction methods are primarily categorized into three types [5,6]: ligand similarity–based methods [7,8], network-based methods [9,10], and structure-based methods [11]. Ligand similarity–based methods predict DTIs by comparing the structural similarity of drugs. These methods are computationally efficient but often overlook complex biochemical properties and molecular characteristics, which may lead to inaccurate predictions [12]. Network-based methods rely on large amounts of high-quality interaction data and are computationally intensive [13-15]. They typically lack structural information about drugs and targets, resulting in poor performance on sparse networks. Structure-based methods usually depend on the three-dimensional structures of target proteins and drugs [16]. However, these methods exhibit limited efficacy for proteins with unknown structures.

To address the above issues, we propose a heterogeneous network (HN) model based on multiview path aggregation for DTI (MVPA-DTI) prediction. MVPA-DTI enhances prediction capability through a multiview feature extraction and fusion mechanism. First, the molecular attention Transformer model is used to extract the three-dimensional spatial structure information of drugs, while the protein-specific LLM Prot-T5 is leveraged to deeply explore the biophysical properties of protein sequences, forming two feature views based on structure and sequence. Subsequently, a HN is constructed by incorporating multisource heterogeneous information from proteins, drugs, diseases, and side effects. This approach effectively integrates the previously defined feature views into the message-passing framework of the HN, thereby forming a comprehensive biological network relationship view. The multiview path aggregation mechanism allows the model to dynamically synthesize feature information from multiple perspectives. By introducing a meta-path aggregation mechanism, the model dynamically synthesizes feature information across different perspectives, capturing underlying drug–target associations at multiple hierarchical levels through both feature-oriented views and biological network relational contexts. During the message-passing process, MVPA-DTI optimizes the weight distribution by incorporating both network topology and biological prior knowledge. By extracting node-level feature embeddings from heterogeneous data, the model is capable of accurately predicting new DTI. Experimental results show that MVPA-DTI outperforms existing advanced methods across multiple evaluation metrics. Additionally, for the voltage-gated inward-rectifying potassium channel KCNH2 target, which is related to cardiovascular diseases, MVPA-DTI was used for candidate drug screening. Among 53 candidate drugs, 38 are predicted to interact with this target (10 of which are already used in clinical treatment). This finding not only validates the effectiveness of MVPA-DTI in predicting DTI but also further demonstrates its application potential and practical value in real drug development. The main contributions of this paper are as follows:

1. We employ a molecular attention Transformer network that extracts three-dimensional structural information from molecular graphs through a physics-informed attention mechanism, establishing a structural feature perspective of the drug.
2. The sequence-level features are extracted from protein sequences using Prot-T5, a protein-specific LLM. The model maps sequence features to functional relevance, establishing a sequence feature perspective of proteins.
3. We integrate the drug structural view and protein sequence view into a multi-entity HN to construct a biological network relationship view. The meta-path information aggregation mechanism captures higher-order interaction patterns among different types of nodes.
4. We propose a multiview path aggregation–enhanced HN model that integrates protein and drug biological knowledge to accurately identify critical drug–target relationships. Benchmark tests show that the model exhibits a significant advantage in prediction performance.

### Related Work

DTI prediction plays a significant role in drug discovery and drug repositioning. With the rapid advancement of deep learning technologies, an increasing number of deep learning–based DTI prediction methods emerge. These methods, particularly those combining big data with deep learning, gradually overcome the limitations of traditional approaches. Based on different prediction strategies, DTI prediction methods are generally categorized into three types [5,6]: ligand similarity–based methods [7,8], network-based methods [9,10], and structure-based methods [11]. In recent years, prediction methods based on LLMs also bring remarkable breakthroughs to the DTI field, opening new pathways for its development.

### Ligand Similarity–Based Methods

In DTI prediction, ligand similarity–based methods estimate potential interactions by comparing the structures or chemical similarity of drug molecules. These methods typically rely on the SMILES (simplified molecular input line entry system) representation or molecular fingerprints of drugs to calculate drug–drug similarities and infer potential target interactions based on these similarities. For instance, Thafar et al [17] proposed the DTiGEMS model, which integrates drug–drug similarities and employs a similarity selection and fusion algorithm to enhance the accuracy of DTI predictions. Additionally, Shim et al [18] introduced a similarity-based convolutional neural network method. This method calculates the outer product of the similarity matrix between drugs and targets and employs a two-dimensional convolutional neural network to capture potential relationships between them. These methods have improved DTI prediction accuracy to some extent, but they often overlook the dynamic interactions and complex spatial structures between molecules. Moreover, ligand similarity–based methods operate under the assumption that similar drug molecules share similar targets. However, this assumption does not always hold, especially when dealing with molecules that exhibit significant structural differences.

### Network-Based Methods

Network-based methods typically rely on large amounts of known DTI data and utilize graph algorithms for modeling. In these methods, drugs and targets are represented as nodes in a graph, while the interactions between them are represented as edges. Additionally, drug–drug similarity networks, protein–protein similarity networks, and known DTI networks are often integrated into a HN. Examples of such approaches include DTI networks, multisimilarity collaborative matrix factorization, and HN models. On this basis, researchers used graph structures to explore potential interaction patterns and promoted the development of tasks such as drug repositioning.

In recent years, with the development of graph representation learning [19,20], network-based methods have achieved deep fusion modeling of structural and semantic information. For example, Zhao et al proposed a regulation-aware graph learning method that enhances drug representation by integrating gene regulation information, thereby improving the prediction effect of drug repositioning [21]. GNN methods mostly rely on the direct adjacency structure of nodes, which easily ignores the rich high-order semantic information and cross-modal associations in biological networks. To this end, some studies have proposed information fusion methods that combine low-order and high-order graph structures. For example, Zhao et al jointly modeled direct neighbor relationships and high-order network path features to effectively enhance the discriminability of drug and target representation [22]. At the same time, in order to more comprehensively model the heterogeneous relationships between multiple entities such as drugs, proteins, and diseases, Zhao et al constructed a heterogeneous information network using semantic paths and attention mechanisms to achieve the integration and discrimination of multimodal information, thereby improving the accuracy and generalization ability of DTI prediction [23]. The iGRLDTI introduced edge weight regulation mechanisms and regularization terms in GNN, further improving the model’s ability to learn potential interactions in heterogeneous biological networks [24].

### Structure-Based Methods

Traditional structure-based methods typically use molecular docking techniques, combined with molecular dynamics simulations, to accurately predict the binding patterns between drugs and targets. With the development of deep learning methods, an increasing number of studies have integrated structural information with neural networks. For example, DeepDTA [25] and DeepDrug3D [26] have enhanced the modeling capability of the drug–target binding process by incorporating three-dimensional structural information of proteins and drugs, further improving prediction accuracy. Although deep learning models can handle structural information, they generally require large amounts of labeled data for effective training, and obtaining high-quality drug–target binding data remains a challenge.

The application of language models is particularly crucial in the absence of molecular structural information. Drawing on techniques from the field of Natural Language Processing, pretrained language models (such as BERT [27] and MolBERT [28]) are used to deeply characterize drug and protein sequence data. By automatically learning latent features and semantic information within sequences, these models enhance the accuracy of DTI predictions. A key advantage of LLMs is their ability to process large-scale, unlabeled data and improve model generalization through transfer learning. For instance, MolBERT [28] and ChemBERTa [29] achieve high-quality molecular representations by pretraining on massive drug molecular datasets. Transformer-based protein language models, such as ProtBERT [30], TAPE [31], and ProtT5 [32], are pretrained on large protein sequence databases. This enables them to capture hierarchical and context-rich sequence representations, enhancing the understanding of protein characteristics. These models can efficiently and accurately characterize proteins using only sequence data, without relying on three-dimensional structural information.

## Methods

### Framework

**Figure 1.**
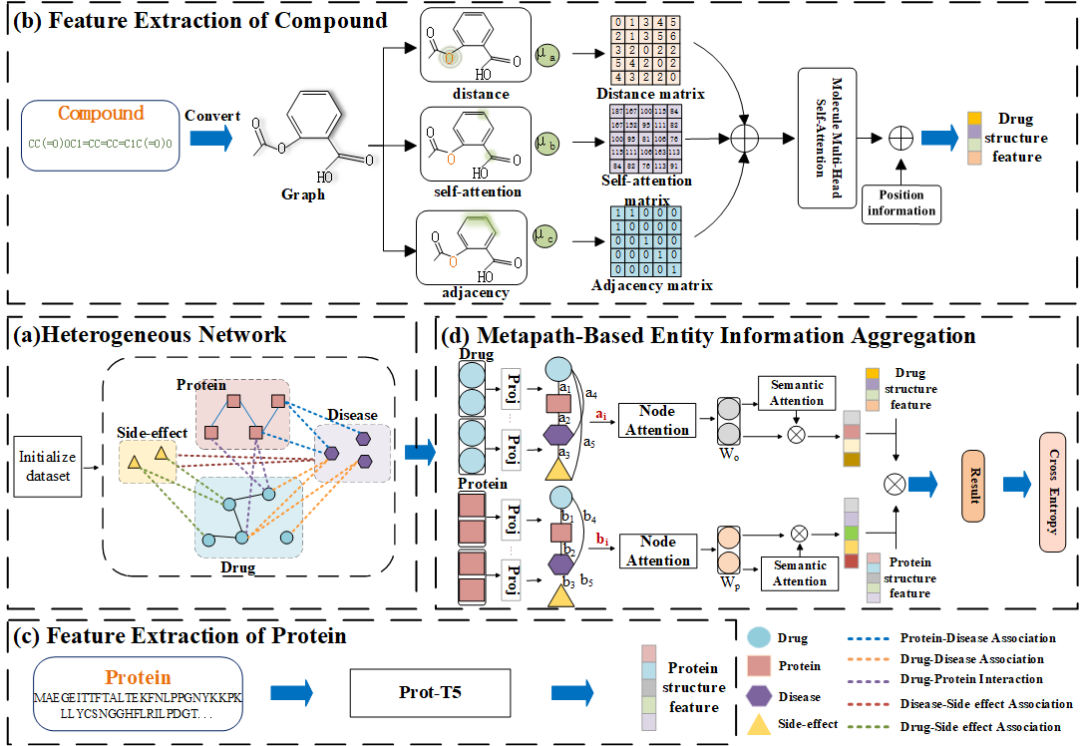
Architecture of the MVPA-DTI model: (A) Construction of the HN; (B) extraction of structural features from the SMILES sequences of drugs to reconstruct drug representations in the HN; (C) extraction of key biophysical properties from protein sequences using Prot-T5 to reconstruct protein representations in the HN; (D) extraction of topological features through the HAN and message-passing mechanism, followed by DTI prediction. DTI: drug–target interaction; HAN: Heterogeneous Graph Attention Network; HN: heterogeneous network; MVPA-DTI: multi-view path aggregation for drug–target interaction; Prot-T5: ProtTrans T5-XL-U50; SMILES: Simplified Molecular Input Line Entry System.

MVPA-DTI combined multiple types of heterogeneous data. It extracted structural and sequential features from drug molecules and protein sequences. It also optimized the weight distribution for information propagation in a heterogeneous graph neural network. As illustrated in Figure 1, the process consisted of four steps: (a) First, an HN is constructed, incorporating drugs, proteins, diseases, and drug side effects. (b) Next, a molecular attention Transformer [33] network is used to extract 3D structural features from the SMILES [34] representations of drugs, which are then assigned to drug nodes. (c) Then, the Prot-T5 model is employed to analyze key biophysical features of protein data, which are subsequently assigned to target nodes in the graph. (d) In the final step, a Heterogeneous Graph Attention Network (HAN) [35] is utilized to analyze network topology, unify node embeddings, determine node importance using meta-paths, and integrate node features through semantic attention. Ultimately, MVPA-DTI predicted potential DTI by optimizing the drug–protein reconstruction loss and the cross-entropy loss.

### Feature View

#### Feature Extraction of Compound Structures

The MVPA-DTI model utilized SMILES sequences as input for drug molecules to learn effective representations of their structural features. Since SMILES sequences shared structural similarities with natural language, contextual information can be effectively leveraged to analyze molecular features. However, traditional methods often struggled to capture long-range atomic relationships when processing molecules. To overcome this limitation, this study employed an improved Transformer architecture.

First, we employed the RDKit toolkit [36] to convert SMILES sequences into molecular graph structures. Since the lengths of SMILES sequences vary across different molecules, a maximum length of 100 is selected to construct reasonable molecular representations. This setting covered at least 90% of the compounds in the dataset. For sequences exceeding this length, truncation is applied, while shorter sequences are padded with zeros to maintain consistent input formatting.

For feature extraction, we employed the Molecule Attention Transformer (MAT) [33] for encoding. Its core idea is to replace the self-attention layer of the traditional Transformer [37] with an enhanced molecular self-attention mechanism. By integrating adjacency information from molecular graphs and interatomic distance information, MAT comprehensively captured the feature representation $S_{drug}$ of drug structures. The attention mechanism integrated interatomic distance and molecular topology. This enhancement allowed the attention distribution to more precisely capture internal molecular relationships. The calculation formula for molecular multihead self-attention is as follows:


(1) $$A^{\left(i\right)}=\left(\mu_{b}\rho \left(\frac{Q_{i}K_{i}^{T}}{\sqrt{d_{k}}}\right)+\mu_{a}g(D)+\mu_{c}A\right)V_{i}$$

where $A\in {\left\{0,1\right\}}^{N_{atoms}\times N_{atoms}}$ represented the adjacency matrix of the molecular graph and $D\in R^{N_{atoms}\times N_{atoms}}$ denoted the distances between atoms. The query, key, and value vector matrices are defined as $Q_{i}=XW_{i}^{q}$, $K_{i}=XW_{i}^{k}$, and $V_{i}=XW_{i}^{v}$, respectively, where *W* represented learnable parameters. In the attention calculation, $\mu_{\alpha }$, $\mu_{b}$, and $\mu_{c}$ corresponded to the weights of different attention components, with specific meanings as follows: $\mu_{\alpha }$ measured the importance of interatomic distances in the attention mechanism; $\mu_{b}$ adjusted the influence of self-attention on the overall attention mechanism; and $\mu_{c}$ controlled the role of the adjacency matrix in the attention computation.

#### Feature Extraction of Protein Structures

The MVPA-DTI model employed Prot-T5 to extract features from protein sequences. By treating sequences as natural language, it used Transformer-based self-supervised learning to capture biological insights. During the data preprocessing stage, protein sequences are treated as text data composed of 20 standard amino acids (along with a few unknown amino acids, such as X). To adapt to the input format of Natural Language Processing tasks, each protein sequence is treated as a token sequence of individual amino acids, with spaces inserted between each amino acid to ensure the Transformer can correctly parse the sequence information. Additionally, to enhance the model’s generalization capability, all uncommon or unresolved amino acid symbols (eg, B, O, U, Z) are mapped to the universal token X, ensuring data consistency.

Prot-T5 is based on the Text-to-Text Transfer Transformer (T5) [38] architecture, which employs an encoder–decoder structure. However, for the task of protein feature extraction, only the encoder part of T5 is utilized to map protein sequences into a high-dimensional feature space. Prot-T5 adopted the Span Masking language modeling strategy, where during training, continuous segments of amino acids in the sequence are randomly masked, and the model is tasked with reconstructing the masked portions based on the context. This enabled the model to learn long-range dependencies and semantic information within protein sequences. As shown in Equation 2, given a protein sequence $S=(S_{1},S_{2},...,S_{n})$, it is first embedded into a high-dimensional space $E\left(S\right)$ and then fed into the Transformer to compute the hidden representations:


(2) H=Transformer(E(S))

Finally, the global representation of the entire sequence is obtained through average pooling, as calculated by the following formula:


(3) $$S_{protein}=\frac{1}{n}\sum_{i=1}^{n}H_{i}$$

where $H_{i}$ represented the hidden states output by the Transformer and $S_{protein}$ served as the global feature of the protein.

### Relational View of Biological Networks

#### Heterogeneous Networks

The HN is described as an undirected graph G = (V, E), where $V=\{\upsilon_{1},\upsilon_{2},...,\upsilon_{n}\}$ represented the set of nodes and E={E(1),E(2),...,E(k)} denoted the set of ktypes of edges, with each $E^{\left(i\right)}=\left\{e_{i1},e_{i2},...,e_{im}\right\}$ representing a specific type of edge.

As shown in Figure 1A, this paper constructed a comprehensive heterogeneous information network, which included drug–drug interactions, drug–protein interactions, drug–disease associations, drug–side effect associations, protein–protein interactions, and protein–disease associations. In the network, entities such as drugs, targets, diseases, and side effects are represented as nodes, while the relationships and interactions between nodes are represented as edges. In the proposed framework, each node belonged to only one type of entity, and all edges are undirected and have nonnegative weights. Node messages are first sent to their first-order neighbors and then propagated to higher-order neighbors through the network edges, a process known as message passing.

The message-passing process in the HN is divided into multiple stages. First, nodes transmitted their information to first-order neighbors. This information is then propagated to higher-order neighbors through network edges, forming more complex information dissemination. Message passing is not limited to directly connected nodes but also includes information transmitted through multihop paths. This enabled the model to integrate features across a broader neighborhood, thereby better capturing long-range dependencies and multilevel network structures.

#### Metapath-Based Entity Information Aggregation

In heterogeneous graph representation learning, our goal is to learn effective feature representations for each node in the HN. However, the challenge of this task lies in not only integrating information from different types of nodes and edges in the heterogeneous graph but also considering the heterogeneous features and content of each node. To address this issue, we employed HAN [35] as the topological feature extraction method for the heterogeneous information network. HAN modeled the structural relationships in the HN through a hierarchical attention mechanism. Specifically, node-level attention learned meta-path-based neighborhood weights and aggregates them to generate semantically specific node embeddings, while semantic-level attention assigned weights across different meta-paths to obtain the optimal task-specific representation, as illustrated in Figure 1D. In terms of meta-path selection, we selected two biologically meaningful paths: D-P-D (Drug–Protein–Drug) and P-D-P-D (Protein–Disease–Protein–Drug). D-P-D is used to capture the semantic relationship between drugs connected by common targets, which helped the model identify drug pairs with similar functions or related potential mechanisms of action, while P-D-P-D further explored the multi-hop indirect connections between proteins formed by disease associations, thereby reflecting a more complex biological network structure. We selected these two meta-paths based on their good semantic interpretability and biological relevance, which helps the model more effectively model the potential interactions between drugs and targets.

After obtaining the node embeddings, we further proceeded with the message-passing process. Assuming the initial node embeddings are defined as $f^{0}:V\to R^{d}$, where $f^{0}(v)$ represented the mapping of node v in the d-dimensional space, the information aggregation process for node v can be expressed as:


(4) $$f_{v}=\sigma \left(\frac{1}{K}\sum_{k=1}^{K}\sum_{u\in N_{v}}\alpha_{uv}W\;f^{0}(u)\right)$$

(5) $$\alpha_{uv}=\frac{exp\left(AconC\left(\alpha^{T}\left[W\;f^{0}\left(u\right)∥W\;f^{0}\left(v\right)\right]\right)\right)}{\sum_{k\in N_{u}}exp\left(AconC\left(\alpha^{T}\left[Wf^{0}\left(u\right)∥W\;f^{0}\left(k\right)\right]\right)\right)}$$

where σ represents a nonlinear activation function, K denotes the number of attention layers, $N_{v}$ represents the set of neighboring nodes of node v, W is the shared weight parameter, $a_{uv}$ is the edge weight computed by the attention mechanism, and AconC(∗) [39] is an adaptive activation function.

Based on obtaining the graph structure representation, we further integrated the structural information of each node to construct the final drug and protein representation. For drug nodes, its representation integrates the meta-path structure features, the original embedding representation, and the molecular structure features, which is specifically expressed as:


(6) $$f_{drug}=L2\;Norm\left(ReLU\left(\left[mate_{DPD},E_{drug}+L2\;Norm\left(S_{drug}\right)\right]W_{0}+b_{0}\right)\right)$$

Similarly, the protein node representation combined its meta-path structure features, original embeddings, and sequence representations and is expressed as:


(7) $$f_{protein}=L2\;Norm\left(ReLU\left(\left[mate_{PDPD},E_{protein}+L2\;Norm\left(S_{protein}\right)\right]W_{0}+b_{0}\right)\right)$$

Among them, $W_{0}$ and $b_{0}$ are learnable linear transformation parameters, which are used to uniformly map the concatenated multiple features to a common low-dimensional semantic space, and implicitly model the importance weights of each feature subspace during the training process to improve the discriminative ability of the final representation.

After completing the representations of drugs and proteins, we used the inner product to calculate the interaction probability between them, as shown in Figure 1D. For a drug node u and a protein node v, their interaction probability is calculated as follows:


(8) $$p^{uv}=\sigma \left({\left(f_{u}\right)}^{T}f_{v}\right)$$

where σ is the sigmoid function and $p^{uv}$ represents the interaction score between node u and node v.

To optimize the model, we employed multiple loss terms for joint training. Among them, the cross-entropy loss is used to supervise the interaction prediction task, defined as follows:


(9) $${£}_{CEL}=\sum_{r\in R}-y_{r}log\left(p_{r}\right)-\left(1-y_{r}\right)log\left(1-p_{r}\right)$$

where R is the set of relationships, $y_{r}$ is the ground truth label of sample r (1 for positive samples and 0 for negative samples), and $p_{r}$ is the predicted probability.

In addition, to enhance the model’s structural perception of node features, we introduced reconstruction losses for drugs and proteins, which are defined as follows:


(10) $${£}_{rec}^{drug}={\left\|{\hat{A}}_{drug}-A_{drug}\right\|}_{F}^{2}$$

(11) $${£}_{rec}^{protein}={\left\|{\hat{A}}_{protein}-A_{protein}\right\|}_{F}^{2}$$

Among them, $A_{drug}$ and $A_{protein}$ are the adjacency matrices of drug and protein nodes in the original HN, respectively. $\hat{A}$ represents the adjacency matrix reconstructed by the model through node embedding and ${\left\|∙\right\|}_{F}$ represents the Frobenius norm. The final optimization target is the weighted sum of three losses:


(12) $$£={£}_{CEL}+\lambda_{1}{£}_{rec}^{drug}+\lambda_{2}{£}_{rec}^{protein}$$

where ${£}_{rec}^{drug}$ and ${£}_{rec}^{protein}$ represent the reconstruction losses for drugs and proteins, respectively, while $\lambda_{1}$ and $\lambda_{2}$ are weighting coefficients used to balance the contributions of different loss terms. Through joint optimization, we can improve the accuracy of DTI prediction while ensuring that the generated feature representations possess robust structural information and discriminative capabilities.

## Results

### Dataset

This study conducted DTI prediction experiments on the Luo dataset [9], which was assembled by Luo et al. To better capture biological characteristics, we incorporated the SMILES sequences of drug molecules and the structural sequence data of proteins into the dataset. Tables1  2 present the relevant statistical information of nodes and edges in the dataset. The dataset comprises six independent drug–protein interaction networks, with all edge weights being binary values.

**Table 1.** Statistics of node information in the dataset.

Node type	Count
Drug	708
Protein	1512
Disease	5603
Side effect	4192

**Table 2.** Statistics of association information between nodes in the dataset.

Edge type	Count	Source
Drug–protein interaction	1923	DrugBank version 3.0 [40]
Drug–drug interaction	10,036	DrugBank version 3.0 [40]
Protein–protein interaction	7363	HPRD Release 9 [41]
Drug–disease association	199,214	Comparative Toxicogenomics Database [42]
Protein–disease association	1,596,745	Comparative Toxicogenomics Database [42]
Drug–side-effect association	80,164	SIDER Release 2 [43]

### Experimental Settings

Building upon the study by Luo et al [9], 90% of the positive and negative samples from the dataset were used to construct the HN and train MVPA-DTI, while the remaining 10% were reserved for testing. In this study, we adopted a data preprocessing process consistent with NeoDTI, including negative sample sampling strategy, drug–target similarity calculation method, and similarity threshold setting to eliminate redundancy. Subsequently, the model’s performance was evaluated through 10-fold cross-validation, with the effectiveness of the method being measured using AUROC and AUPR metrics.

MVPA-DTI consists of three modules: Prot-T5, MAT, and HAN, with the parameter settings detailed in Table 3. During the training process, we employed the Adam optimizer to update the network weights.

**Table 3.** Parameter settings in the experiment.

Parameter	Value
Number step	3000
Batch size	128
Learning rate	0.001
Dropout rate	0.1
HAN^a^ input size	1024
MAT^b^ multi-head attention number	16
MAT stack number	8

a HAN: Heterogeneous Graph Attention Network.

b MAT: Heterogeneous Graph Attention Network.

### Comparison With Prior Work

In this study, to better simulate real-world scenarios, we randomly sampled negative samples while retaining all positive samples, setting the ratio of positive to negative samples at 10:1. This ratio aims to reflect the imbalance between positive and negative samples in the real world. Subsequently, we compared the performance of MVPA-DTI with several benchmark models to evaluate its effectiveness in DTI prediction tasks; they are briefly described as follows:

- MSCMF [44]: MSCMF effectively predicts DTI by utilizing multiple similarity matrices of drugs and targets through collaborative matrix factorization.
- HNM [45]: HNM integrates disease, drug, and target information via a HN, aiming to enhance the efficiency and accuracy of drug repositioning.
- DTINet [9]: DTINet predicts interactions between new drugs and targets by integrating heterogeneous data and learning low-dimensional feature representations.
- NeoDTI [46]: NeoDTI automatically generates feature representations by integrating diverse information from HN.
- EEG-DTI [47] : EEG-DTI is an end-to-end learning framework based on heterogeneous graph convolutional networks, capable of effectively learning features from multiple biological entities.
- SHGCL-DTI [48]: SHGCL-DTI combines semi-supervised learning with graph contrastive learning, enhancing the model’s adaptability.

The performance of MVPA-DTI was compared with various models, and the experimental results are shown inTable 4. MVPA-DTI achieves the best performance, demonstrating significant improvement over other DTI models. It achieves an AUPR of 0.901, which is 1.7% higher than the second-best method, and an AUROC of 0.967, representing a 0.9% improvement over SHGCL-DTI. MSCMF employs matrix transformation to optimize prediction results through network inference and the topological structure of data. However, compared to the recent outstanding performance of deep learning methods, MSCMF does not fully exploit the latent information in data matrix embeddings or the features of adjacent nodes, thereby limiting its predictive performance. HNM does not adopt mainstream heterogeneous data embedding methods for feature representation and information integration, resulting in insufficient generalization ability and lower prediction accuracy. In contrast, DTINet, NeoDTI, and EEG-DTI further extract hidden features by combining matrix transformation with neural networks, enabling more accurate modeling of node relationships and improving prediction performance. SHGCL-DTI employs a graph contrastive learning strategy to capture the structural information of heterogeneous graphs. This is achieved by enhancing the similarity of positive sample pairs and reducing the similarity of negative sample pairs. However, SHGCL-DTI fails to fully utilize the rich semantic information and complex interaction patterns in heterogeneous graphs, presenting certain limitations when processing biological data.

**Table 4.** The AUPR and AUROC evaluation results for each model.

Model	AUPR^a^	AUROC^b^
HNM^c^	0.579	0.834
MSCMF^d^	0.603	0.856
DTINet^e^	0.818	0.916
NeoDTI^f^	0.855	0.943
EEG-DTI^g^	0.847	0.952
SHGCL-DTI^h^	0.884	0.958
MVPA-DTI (ours)^i^	0.901	0.966

a AUPR: area under the precision–recall curve.

b AUROC: area under the receiver operating characteristic curve.

c HNM: heterogeneous network model.

d MSCMF: multiple similarities collaborative matrix factorization.

e DTINet: drug–target interaction prediction network.

f NeoDTI: neural integration of neighbor information for DTI prediction.

g EEG-DTI: end-to-end graph for drug–target interaction prediction

h SHGCL-DTI: semi-supervised heterogeneous graph contrastive learning for drug–target interaction prediction.

i MVPA-DTI: multi-view path aggregation for drug–target interaction.

Existing methods fail to fully exploit critical biochemical information in drug molecular structures and protein sequences, potentially leading to information loss during the node embedding process. In contrast, MVPA-DTI integrates a graph attention network that incorporates all types of adjacent nodes, enhancing the extraction of composite structural information. Furthermore, it employs HAN to model the potential relationships between different types of nodes. Additionally, the method dynamically fuses features extracted from sequences and graph to update the embedding representations of drug and protein nodes. Through this process, it progressively strengthens the weight of node information and optimizes the feature representation of different types of nodes, thereby improving the predictive accuracy.

### Robustness Experiment

To further validate the stability of MVPA-DTI, considering the potential presence of redundant information in the dataset, additional experiments were conducted to evaluate the model’s predictive performance. First, we removed some samples from the dataset, including DTIs with similar drugs or targets and DTIs involving drugs with similar drug interactions. The details of the removed data are provided in Table 5.

**Table 5.** Number of drugs, targets, and DTIs included in the redundant data.

Redundant data	Drug number	Target number	DTI number
DTI of similar drugs or targets	146	78	955
DTI of drugs with similar side effects	17	0	51

a DTI: drug–target interactions.

After removing similar drugs and targets, as shown in Table 6, MVPA-DTI achieves a 15.9% improvement over the second-best method, NeoDTI. Meanwhile, Table 6 also demonstrates that after removing drugs with similar side effects, MVPA-DTI outperforms the second-best method by 3.1%.

**Table 6.** AUPR performance of each model after adjustment of the dataset.

Model	AUPR^a^		
	DTIs^b^ with similar drugs and targets were removed	DTIs with drugs with similar side effects were removed	Trained on non-unique dataset and tested on unique dataset
HNM^c^	0.547	0.581	0.233
MSCMF^d^	0.265	0.593	0.206
DTINet^e^	0.611	0.803	0.389
NeoDTI^f^	0.694	0.848	0.432
EEG-DTI^g^	0.686	0.846	0.431
SHGCL-DTI^h^	0.617	0.729	0.443
MVPA-DTI^i^	0.849	0.873	0.46

a AUPR: area under the precision–recall curve.

b DTI: drug–target interaction.

c HNM: heterogeneous network model.

d MSCMF: multiple similarities collaborative matrix factorization.

e DTINet: drug–target interaction prediction network.

f NeoDTI: neural integration of neighbor information for DTI prediction.

g EEG-DTI: end-to-end graph for drug–target interaction prediction.

h SHGCL-DTI: semi-supervised heterogeneous graph contrastive learning for drug–target interaction prediction.

i MVPA-DTI: multi-view path aggregation for drug–target interaction.

The experimental results indicate that although the model’s performance declined after removing a significant portion of specific DTI data, MVPA-DTI still maintains the best AUPR, demonstrating its strong robustness. Additionally, this study treats drug–protein interactions as a specific case for experimentation. To objectively evaluate the predictive capability of MVPA-DTI, both special drug–target relationships and conventional drug–target relationships were separately processed. Specifically, the model was first trained on a dataset in which the relationships between drugs and proteins were nonunique and then tested on a dataset with unique interactions. As shown in Table 6, MVPA-DTI significantly outperforms the second-best method, with a 1.7% improvement in AUPR. This result suggests that MVPA-DTI exhibits stronger generalization ability in predicting DTI.

### Ablation Experiment

To verify the contribution of each module in MVPA-DTI, we conducted ablation experiments. The experimental results are presented in Table 7, where ProSF represents the protein sequence feature extraction module and DruSF denotes the drug compound structure feature extraction module.

**Table 7.** Evaluation of the impact of MVPA-DTI modules on performance.

Method	AUROC^a^	AUPR^b^	*F*_1_-score	MCC^c^
w/o^d^ ProSF^e^	0.963	0.886	0.839	0.828
w/o DruSF^f^	0.964	0.891	0.840	0.827
w/o ProSF and DruSF	0.957	0.875	0.813	0.804
protBert-MVPA-DTI^g^	0.964	0.89	0.831	0.822
MVPA-DTI^h^	0.966	0.901	0.848	0.839

a AUROC: Area Under the Receiver Operating Characteristic curve.

b AUPR: Area Under the Precision-Recall Curve.

c MCC: Matthews Correlation Coefficient.

d w/o denotes corresponding module was removed.

e ProSF: protein sequence feature extraction module.

f DruSF: drug compound structure feature extraction module.

g protBert-MVPA-DTI replaces the ProSF module with ProtBert processing.

h MVPA-DTI: multi-view path aggregation for drug-target interaction.

#### The Effectiveness of ProSF

As shown in Table 7, the ProSF module improves the model performance by 0.3% in AUROC and 1.5% in AUPR. ProSF effectively extracts deep semantic information from proteins, capturing features related to key physicochemical properties such as secondary structure and solubility, thereby enhancing the model’s predictive capability.

#### The Effectiveness of DruSF

As shown in Table 7, the DruSF module enhances the model’s performance, with improvements of 0.2% and 1.0% in AUROC and AUPR metrics, respectively. This enhancement is attributed to DruSF’s ability to thoroughly explore the structural features of drugs, thereby assigning higher weights to drugs during the message-passing process and further optimizing the prediction outcomes.

The experimental results demonstrate that the MVPA-DTI model exhibits high effectiveness in extracting the biological structural features of drugs and proteins. By deeply exploring the chemical structures, physical properties, and biological functions of drugs and proteins, the model can accurately capture the complex interaction relationships between them, thereby enhancing DTI prediction performance. Further analysis reveals that protein structural information plays a more critical role than drug structural information in the prediction process. This phenomenon may be attributed to the protein-specific LLM Prot-T5, which more effectively captures evolutionary conservation and functional relevance in protein sequences, thereby providing more discriminative feature representations for DTI prediction.

## Discussion

### Case Study

In evaluating the practical application of MVPA-DTI, we predicted 53 candidate drugs targeting the voltage-gated inwardly rectifying potassium channel KCNH2 (hERG). The KCNH2 channel, a critical protein in cardiac electrophysiology, plays a central role in regulating ventricular repolarization. Dysfunction or dysregulation of this channel delays ventricular repolarization, manifesting as QT interval prolongation on electrocardiograms. This phenomenon has been extensively documented to correlate with the pathogenesis of various cardiovascular diseases [49]. The results demonstrate that 38 out of the 53 candidate drugs exhibited potential interactions with KCNH2. Among these, 10 drugs have been experimentally validated in published studies to interact with the KCNH2 channel. Although the remaining candidates have not yet been experimentally confirmed, their established associations with cardiovascular pathologies suggest potential therapeutic relevance in cardiovascular disease management. Studies have demonstrated that procainamide interacts with the KCNH2 channel, primarily by inhibiting its function. This inhibition prolongs the duration of potassium ion efflux, leading to QT interval prolongation [50]. Nicotine, as a blocker of the KCNH2 channel, significantly affects the electrophysiological properties of the heart. This interaction may have important clinical implications for assessing the impact of nicotine on cardiac health [51]. Ranolazine is considered to have potential therapeutic effects, as it improves cardiac electrophysiological abnormalities caused by genetic variations by modulating the function of the KCNH2 channel [52].

**Figure 2.**
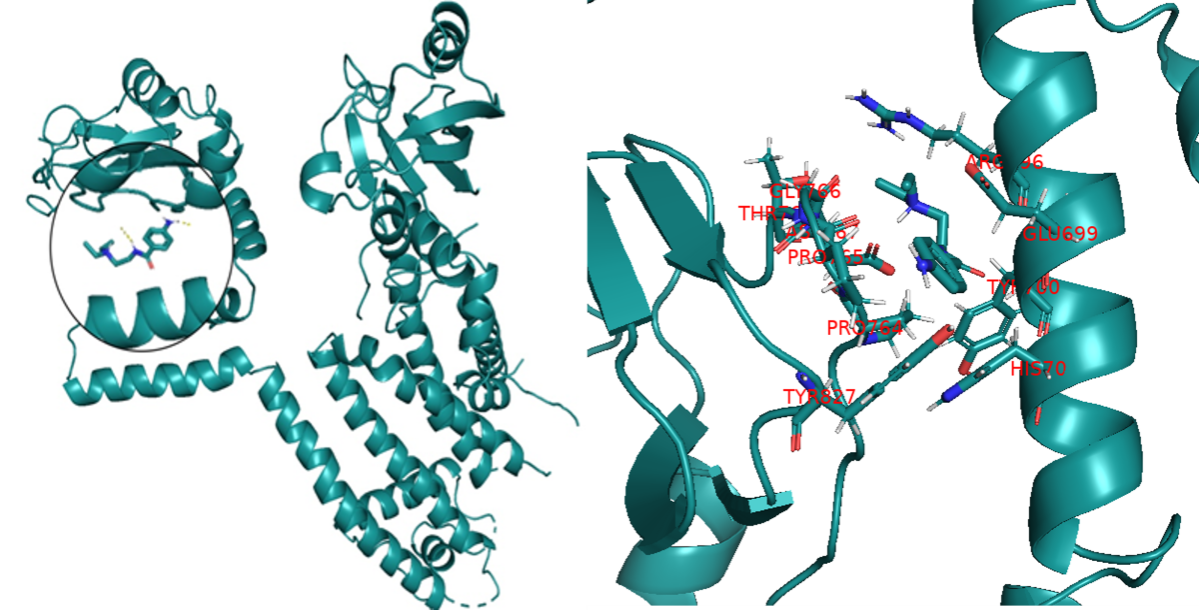
Visualization of the interaction between procainamide and the KCNH2 channel. KCNH2: Potassium Voltage-Gated Channel Subfamily H Member 2.

**Figure 3.**
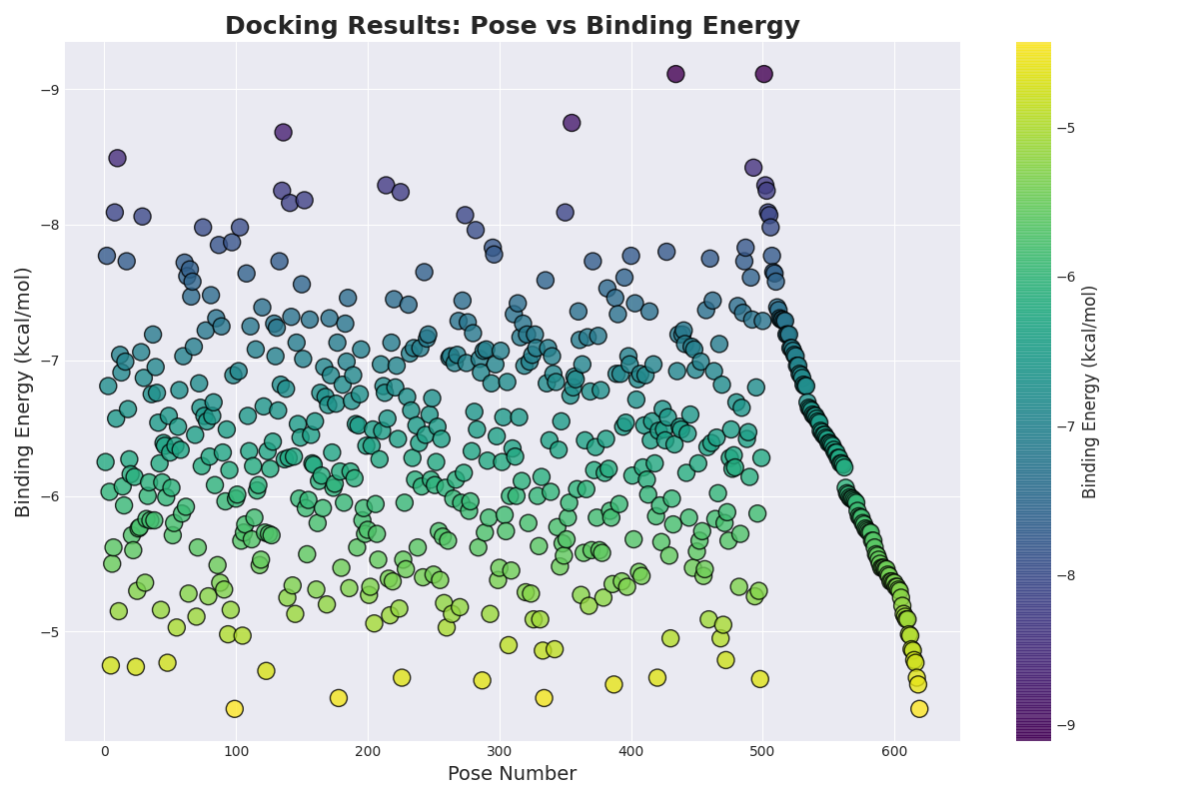
Binding energy distribution of procainamide docking with KCNH2. KCNH2: Potassium Voltage-Gated Channel Subfamily H Member 2.

In this study, AutoDock was used to perform molecular docking simulations on the interaction between procainamide and KCNH2 channel protein. As shown in Figures2  3, procainamide can stably bind to the central cavity region of the KCNH2 channel protein and is embedded in a hydrophobic pocket surrounded by the S6 helix and the lamellar structure. The binding site involves multiple key amino acid residues, including THR 768, TYR 827, etc. These residues have been widely reported in the literature as core sites for regulating drug binding and gating behavior of KCNH2 channels. These binding residues are visually annotated in Figure 2. Binding mode analysis showed that hydrophobic interactions and potential hydrogen bonds were formed between procainamide and the above residues, which may interfere with the open state of the channel and affect its function. As shown in Figure 3, binding energy analysis showed that procainamide interacted strongly with KCNH2, and the binding energy of some conformations was as low as –9 kcal/mol, indicating that it has high binding stability and potential biological activity.

### Conclusions

This study proposes a novel DTI prediction method MVPA-DTI, which extracts key biological features from protein and drug sequences and reconstructs them into a heterogeneous graph, enabling the model to capture the most critical biological information during each iteration, thereby optimizing the weight assignment for drugs and targets. Experimental results demonstrate that MVPA-DTI outperforms existing methods across multiple benchmark tests. Although MVPA-DTI effectively captures DTI, the biological mechanisms underlying these interactions are complex, involving multiple factors, which are not yet fully considered by MVPA-DTI. Future improvements should focus on in-depth exploration of the biological details of DTI to enhance prediction accuracy and applicability. To enhance the ability to model the complex relationship between drugs and targets, in the future, it is possible to consider introducing new graph neural network structures such as FCGCN [53] into the drug–target graph modeling process to more effectively integrate molecular structure, pharmacological properties, and network topology information.

## Abbreviations

DruSF: drug compound structure feature extraction module
DTI: drug-target interaction
HAN: Heterogeneous Graph Attention Network
HN: Heterogeneous Network
LLM: large language model
MAT: Molecule Attention Transformer
ProSF: protein sequence feature extraction module.
